# Lithium Iron Phosphate and Layered Transition Metal Oxide Cathode for Power Batteries: Attenuation Mechanisms and Modification Strategies

**DOI:** 10.3390/ma16175769

**Published:** 2023-08-23

**Authors:** Guanhua Zhang, Min Li, Zimu Ye, Tieren Chen, Jiawei Cao, Hongbo Yang, Chengbo Ma, Zhenggang Jia, Jiwei Xie, Ning Cui, Yueping Xiong

**Affiliations:** 1 Queen Mary University of London Engineering School, Northwestern Polytechnical University, Xi’an 710100, China; 2 School of Management, Northwestern Polytechnical University, Xi’an 710100, China; 3 School of Mechanics Civil Engineering and Architecture, Northwestern Polytechnical University, Xi’an 710100, Chinatwilight@mail.nwpu.edu.cn (C.M.); 4 School of Aeronautics, Northwestern Polytechnical University, Xi’an 710102, China; 5 School of Materials Science and Engineering, Harbin Institute of Technology, Harbin 150001, China; 6 School of Life Science, Northwestern Polytechnical University, Xi’an 710100, China; 7 School of Chemistry and Chemical Engineering, Harbin Institute of Technology, Harbin 150001, China

**Keywords:** lithium iron phosphate (LFP), nickel–cobalt–manganese (NCM), cathode materials, power battery, cycle life, attenuation mechanism

## Abstract

In the past decade, in the context of the carbon peaking and carbon neutrality era, the rapid development of new energy vehicles has led to higher requirements for the performance of strike forces such as battery cycle life, energy density, and cost. Lithium-ion batteries have gradually become mainstream in electric vehicle power batteries due to their excellent energy density, rate performance, and cycle life. At present, the most widely used cathode materials for power batteries are lithium iron phosphate (LFP) and Li_x_Ni_y_Mn_z_Co_1−y−z_O_2_ cathodes (NCM). However, these materials exhibit bottlenecks that limit the improvement and promotion of power battery performance. In this review, the performance characteristics, cycle life attenuation mechanism (including structural damage, gas generation, and active lithium loss, etc.), and improvement methods (including surface coating and element-doping modification) of LFP and NCM batteries are reviewed. Finally, the development prospects of this field are proposed.

## 1. Introduction

Since the invention of lead–acid batteries in the 1880s, batteries have been closely related to the development of transportation. The development of power batteries has become crucial to the development and popularization of electric vehicles that meet the ‘dual carbon’ requirements of ‘carbon peaking and carbon neutrality’. Nickel–Cadmium batteries, nickel–metal hydride batteries, and lead–acid batteries have all been used for electric vehicles. However, due to the inadequacy of these batteries’ multiplying performance, capacity, and cycle life, they cannot meet the requirements of electric vehicles and have been gradually eliminated. Due to their high energy density and excellent cycle performance, lithium-ion batteries (LIBs) have dominated the portable electronics market since their commercialization 25 years ago and have played an increasing role in the global commercialization of electric vehicles in the previous decade. First-generation LIBs with LiCoO_2_ (LCO) as the cathode have an operating voltage of 3.7 V and a theoretical specific discharge capacity of 140 mAh/g [1]. However, the high price of cobalt metal and the tendency for thermal runaway have led to the development and use of cathode materials with little or no cobalt [2]. Due to their stability and safety performance, LIBs have become the main technological driver of the electric vehicle revolution (electric vehicles replacing vehicles based on internal combustion engines) [3].

Research concerning high-energy lithium cathodes mainly consists of the following three directions: (1) the spinel structure represented by LiMn_2_O_4_ [4], (2) the layered transition metal oxide represented by Li_x_Ni_y_Mn_z_Co_1−y−z_O_2_ (NCM) [5], and (3) the olivine structure represented by lithium iron phosphate (LFP) [6]. In addition, the requirement for higher specific energy and energy density has led to interest and efforts in developing rechargeable batteries with lithium metal anodes [7] and solid electrolytes (polymers and ceramics) [8], although the lithium ion conductivity in the solid electrolyte is lower than that in the liquid electrolyte [8]. In addition, it has to be pointed out that although some research results show good electrochemical properties in the laboratory, their poor repeatability and complex processes hinder their large-scale application.

Here, we review the attenuation mechanism and modification strategies concerning the use of LFP and NCM as power batteries. In detail, the modification of LFP and NCM via lattice doping and surface coating is discussed in order to obtain a high-capacity retention rate and stable operating voltage. In addition, we also discuss the safety evaluation of LFP and NCM and their applications in solid-state batteries. Finally, we provide a feasible solution to the degradation of battery life.

## 2. Properties of LFP and NCM

The performance and cost of electric vehicles are largely determined by power batteries. The application cost of power batteries is mainly determined by their cycle life. As shown in Table 1, considering that the electric vehicle is charged once every two days, lead–acid batteries can be used for two years; nickel–metal hydride batteries can be used for four years; while LIBs can be used for even eight years. Furthermore, the energy density of the LIBs is 2–3 times that of lead–acid batteries and nickel–metal hydride batteries, reducing the dead weight of the battery while extending the driving range. 

The cathode materials of LIBs include LFP, NCM, lithium cobaltate (LCO), and lithium manganate (LMO) etc. As shown in Table 1, LFP shows extremely high cycle life and a stable voltage platform, which can effectively reduce battery weight and ensure the acceleration ability of electric vehicles. NCM also exhibits high energy density, cycle life, and Li^+^ diffusion ability. In particular, NCM displays a good rate performance during high-rate discharge, which can support the high-power charging demand of power batteries and the high current required for electric vehicle acceleration. However, due to the increasing prices of precious metals in recent years, the popularity of NCM batteries has been limited. LCO has a high specific capacity and battery voltage of 4.2 V, leading to high energy density [9]. However, due to the phase transition of LCO during lithium removal and insertion, Co is unstable and prone to dissolution under high-voltage platforms. The side reactions between dissolved Co and electrolyte or anode can lead to serious safety concerns and limit the application of LCO. In addition, the low diffusion coefficient and conductivity of Li^+^ in LMO severely limit its application in high-rate discharge [10]. In summary, LFP and NCM show advantages in terms of cost-effectiveness and performance, making them the mainstream in electric vehicle power batteries.

**Table 1:** Comparison of common power battery parameters.

Battery Properties	Lead–Acid Battery [11]	Nickel–Metal Hydride Battery [12]	Lithium-Ion Battery		
			LFP [13]	NCM [14]	LCO [15]
Voltage (V)	2	1.2	3.3	3.6	3.7
Specific energy (Wh·kg^−1^)	35–45	50–80	130–140	160–220	135–150
Li^+^ diffusion coefficient (cm^2^·s^−1^)	/	/	10^−16^–10^−14^	10^−11^–10^−10^	10^−12^–10^−11^
cycle life (times)	300–500	500–1000	2000–6000	1500–2000	500–1000

## 3. Lithium Iron Phosphate (LFP) Battery

### 3.1. Structure and Properties of LFP

**Figure 1:**
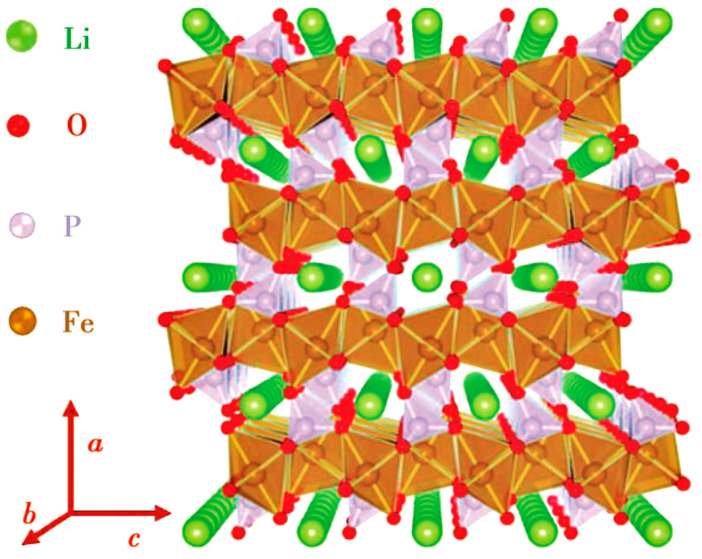
LFP crystal structure showing one-dimensional diffusion channel of Li^+^ [16].

LFP has an olivine crystal structure [16], which transforms into the FePO_4_ (FP) phase during the charging process. Due to the similar crystal structure of the two phases, the volume change of the crystal cell before and after discharge is only 6.81%. Therefore, the internal stress formed in LFP is relatively small during the charging/discharging processes [17]. In addition, the P-O chemical bond is strongly stable, and thus the oxygen in the lattice is not easily lost. The stable crystal structure and very small internal stress make LFP extremely stable in terms of structure, with a cycle life reaching more than 2000 times the length and high safety [18]. Unfortunately, the Li^+^ diffusion coefficient and conductivity in LFP are very low due to two reasons: (1) in the LFP lattice (see Figure 1), electrons propagate through the transition metal layer, and each FePO_4_ is separated by an oxygen atom, with only one common vertex oxygen acting as the connection point. Therefore, LFP is almost an insulator, with an electron conductivity of only 10^−9^ s/cm; (2) the diffusion channel of Li^+^ is one-dimensional, and the PO_4_ tetrahedron hinders the diffusion of Li^+^ in the *c*-axis direction, which increases the diffusion resistance of Li^+^ between LFP and FP phases.

### 3.2. Life Attenuation Mechanisms of LFP Batteries

**Figure 2:**
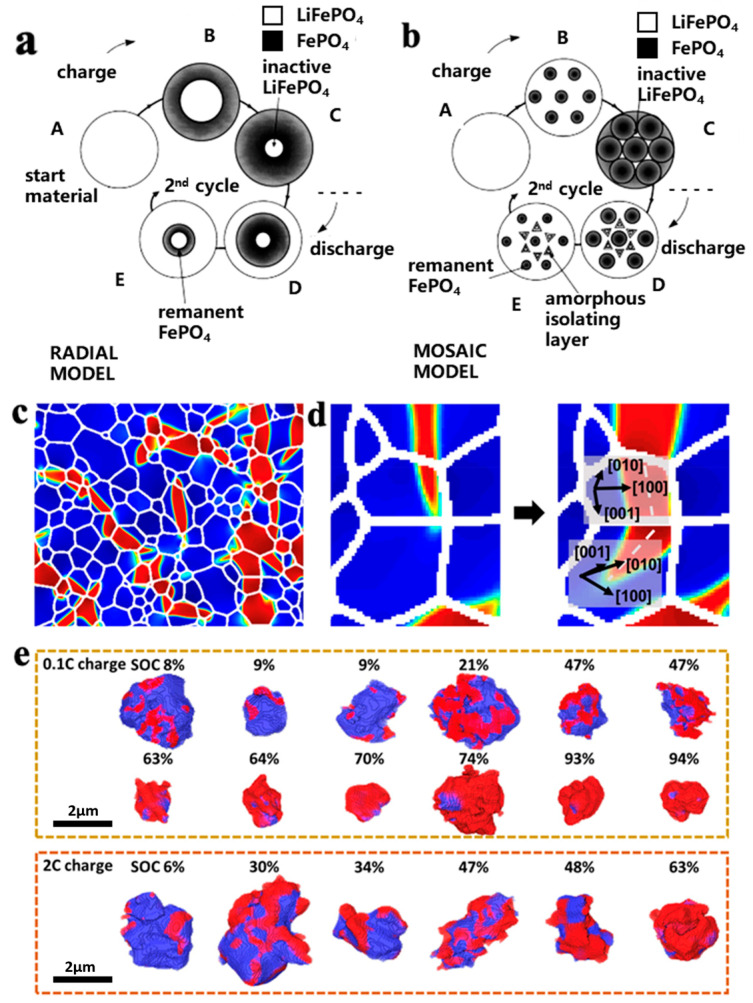
Schemes of (**a**) core–shell and (**b**) mosaic models for Li^+^ extraction/reinsertion in LFP particles [20], (**c**) FP phase in the form of fine filaments during lithium removal [21], (**d**) FP filament changes direction when expanding to another particle [21], (**e**) polarization of LFP particles during charging at rates of 0.1 C and 2 C [21].

Padhi et al. [19] proposed a core–shell model for the lithium extraction/reinsertion of LFP particles, as shown in Figure 2a. The LFP/FP interface moves inward when lithium is extracted from the cathode particles to form FePO_4_. Due to the low conductivity and Li^+^ diffusion coefficient of LFP, the LFP inside the particles cannot fully extract Li^+^ and electrons, which can easily lead to capacity loss. Andersson et al. [20] used the core–shell model and proposed a mosaic model, as shown in Figure 2b. In this model, the extraction and insertion of lithium can occur at random sites in the particles. The lithium extraction regions form inactive Li^+^ isolated regions when they collide with each other, and during discharge, Li^+^ is re-embedded into the LFP. The core FP is not fully converted, and some LFP is enveloped by FP, which can also cause capacity loss. Compared with the core–shell model, the mosaic model proposes that the lithiation and delithiation of LFP are not uniformly embedded and removed from the particle surface in the usual sense but exhibit significant heterogeneity. The above two models may explain the reason for capacity decay: (1) the deactivation of some lithium-rich LFP particles; (2) some FP in the particle core is not fully converted, resulting in the loss of active lithium. The factors causing the above two types of capacity attenuation can be attributed to the low Li^+^ diffusion rate and low electron conductivity of LFP and FP phases, which limit the conversion of the two phases inside the particles. In recent years, researchers have improved the cycling stability and rate performance of LFP by reducing its particle size. The reduction in particle size enhances the migration ability of Li^+^ in the LFP and FP phases and promotes the mutual transformation of the two phases.

Although the above model explains the mechanism of capacity loss in LFP well, it does not take into account the properties of one-dimensional lithium channels in olivine-structured LFP. Fan et al. [21] combined transmission X-ray microscopy (FF-TXM) with X-ray absorption near-edge structure (XANES) spectroscopy and tomography reconstruction to analyze the phase distribution of LFP secondary particles, as shown in Figure 2c. It was found that the FP domain in the particles exhibits a filamentary feature with a large transverse-to-longitudinal ratio. Since the [100] direction has the maximum lattice mismatch of 5% ([001] 1.9%, [010] 3.6%) between LFP and FP, the FP phase tends to grow in the vertical [100] direction because it can minimize the strain energy. When the FP phase extends to the grain boundary, the growth direction of the FP phase tends to change to the path with minimum resistance, as shown in Figure 2d. The characteristic of the FP phase distributed in a fine filament during the LFP lithium removal process further indicates that the formation of the FP phase in the mosaic model is not random. Yang et al. [18] investigated the polarization of LFP/FP phases under different states of charge (SOC) of LFP secondary particles, as shown in Figure 2e. When SOC < 10%, the FP phase tends to form on the surface. As SOC increases, the growth and polarization degree of the FP phase on the surface increases and gradually extends to the interior of the particles, which is consistent with the mosaic model. When SOC > 60%, the FP phase condenses with each other to form a core–shell structure, and the polarization degree of the 2 C high-rate discharge is greater than that of the 0.1 C low-rate discharge. This indicates that the embedding of lithium is accompanied by an obvious polarization phenomenon, and polarization intensifies during high-rate discharge. A large polarization voltage will not only lead to the appearance of lithium deposition but also reduce the operating voltage, resulting in a decrease in cycle performance and energy density. In addition, due to a 6.8% volume difference between LFP and FP, internal stress is generated and structural collapse may occur, resulting in the pulverization of the cathode particles.

### 3.3. Modification Methods for LFP Batteries

Compared with other electrode materials, LFP has good structural stability and excellent cycle life. Sun et al. [17] found that no significant structural damage occurred in the LFP electrode after cycling 1500 times at a rate of 0.5 C. However, the low conductivity and Li^+^ diffusion coefficient of LFP reduce the rate performance of LFP batteries, generate polarization phenomena, and cause a loss of active substances. Therefore, current modification methods of LFP mainly focus on improving the conductivity and Li^+^ diffusion coefficient of LFP. The common methods include reducing particle size, coating, and doping. Among them, reducing the particle size can effectively improve the migration ability of Li^+^ in LFP. However, reducing the particle size may reduce the compacted density, increase the specific surface area, and trigger side reactions between the electrode and electrolyte. Therefore, reducing the particle size of LFP has to be combined with coating or doping.

#### 3.3.1. Metal Doping Modification of LFP

Element doping is considered to be the most effective method to improve the electric conductivity and ion diffusion rate of LFP. The element doping usually occurs at the Li or Fe sites. Appropriate element doping has the following positive effects on LFP: (1) expanding the diffusion channel of Li^+^, reducing the bond energy of Li-O, which is beneficial for the insertion and extraction of Li^+^; (2) increasing the density of Li vacancies, which is beneficial for the diffusion of Li^+^; and (3) reducing the bandgap width of the two phases to increase electric conductivity. Yan et al. [22] used a solid-state method to prepare Mg-doped LFP/C samples and found that the discharge capacity of LFP did not change very much before and after doping at low current, while the discharge capacity of doped LFP was much better than that of undoped samples at high current. This is because Mg doping improves the intrinsic conductivity of the electrode, resulting in a significant increase in cycle life. The capacity retention rate of 1000 high-rate discharges at 10 C can reach 80%, while the capacity retention rate of undoped LFP is only 49% [23]. Doping can limit grain growth, shorten Li^+^ diffusion distance, and improve electronic conductivity. Usually, impurity atoms tend to replace each other with elements with similar radii at the doping sites, but not all doped atoms can enter the LFP lattice. Doped atoms that do not enter the lattice may affect the diffusion of Li^+^, so it is necessary to control the appropriate doping amount [24]. Tao et al. [25] doped the Fe site using Nb via solid-state sintering. As the ion radius of Nb^5+^ is smaller than Fe^2+^, the bond length shortens, leading to enhanced crystalline stability. Moreover, due to the different valence states of Nb^5+^ and Fe^2+^, lattice defects and vacancies are introduced after doping, which improves the conductivity of the material. The capacity of LFP before doping is 137.49 mAh·g^−1^, with a capacity retention rate of 95.44% after 100 cycles at a rate of 1 C. After Nb^5+^ doping, the capacity increased to 169.87 mAh·g^−1^, and had a much-improved capacity retention rate of 99.03% after 100 cycles at a rate of 1 C.

#### 3.3.2. Nanosizing and Carbon Coating of LFP Particles

**Figure 3:**
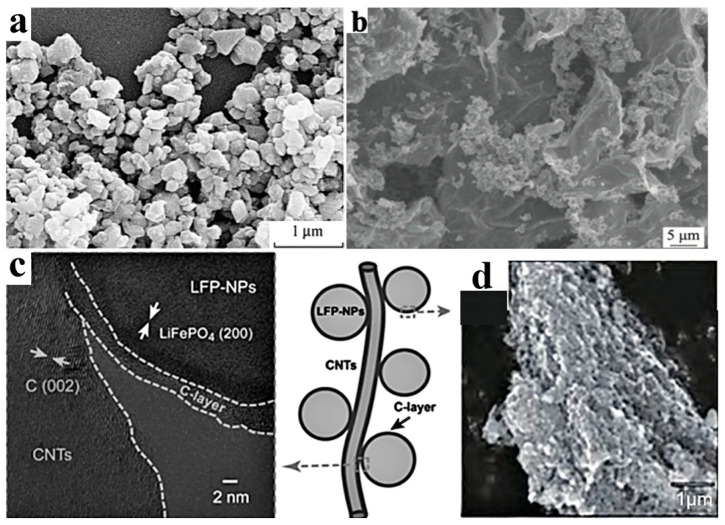
Carbon coating on LFP particles. (**a**) SEM morphology of LiFePO_4_/C [16], (**b**) SEM morphology of LiFePO_4_/activated carbon/graphene [22], (**c**) TEM morphology of LiFePO_4_/C/CNT structure [27], (**d**) SEM image of LiFePO_4_/graphite [16].

The Li^+^ diffusion coefficient of LFP is only 10^−9^~10^−10^ cm^2^·s^−1^, while the Li^+^ diffusion coefficient of the most commonly used graphite is 10^−6^ cm^2^·s^−1^. The Li^+^ diffusion coefficient of LFP, which is close to that of insulators, severely restricts its rate performance. Nanosizing and surface coating are effective modification methods for LFP particles. As the ion conductivity and electronic conductivity at the particle interface are larger than those inside the particle, the diffusion distance inside the LFP particle can be shortened. Thus, nanosizing can significantly improve the conductivity and ion diffusion rate. However, the creation of nanoparticles significantly increases the specific surface area of the particle in contact with the electrolyte, which often leads to serious electrode–electrolyte interface side reactions. Carbon coating can alleviate the problem of interface side reactions. Duan et al. [26] prepared electrode materials coated with dense carbon by mixing LFP particles with an average size of 42 nm with a glucose solution. The specific capacity of the prepared battery was 152 mAh·g^−1^, which was slightly lower than the theoretical value of 170 mAh·g^−1^, but it showed a higher cycle stability and rate performance (capacity loss only 0.3–1.1% after 1000 cycles at 10 C rate). The common coating materials for LFP particles include carbon materials, metals, and metal oxides, ion conductive materials, etc. Considering the increasing requirements for cost-effectiveness and manufacturing feasibility in relation to the processes of power batteries, carbon material coating seems to be the most reliable solution. According to the coated carbon source, the coating method can be divided into inorganic carbon sources (e.g., carbon black, carbon nanotubes, graphene, etc.) and organic carbon sources (e.g., sucrose, glucose, starch, etc.). According to the morphology of coated carbon, the coating method can be divided into one-dimensional carbon (carbon fibers, carbon nanotubes), two-dimensional carbon (graphene), and three-dimensional carbon (carbon nanotube array, graphene skeleton), as shown in Figure 3. It should be pointed out that the metal oxide layer can achieve the neutralization of HF, reduce acidity, and weaken transition metal migration. ZnAl_2_O_4_@LFP, designed by Moustafa et al. [14], improves the ionic conductivity and polarization of LFP, resulting in more lithium ions embedded in LFP. Thus, the ZnAl_2_O_4_@LFP battery shows a high-capacity retention of ~66% after 50 cycles [14].

It is crucial to select an appropriate method to form a uniform carbon coating layer on the surface of LFP based on the coated carbon source. Common coating methods include the following: (1) non-in situ carbon coating (preparing LFP first and then coating) and (2) in situ carbon coating, which refers to the addition of organic carbon sources (sucrose, glucose, citric acid, etc.) to the LFP precursor, simultaneously forming a carbon layer and LFP. The preparation methods include solid-state preparation, wet preparation, gas-phase preparation, etc. In situ carbon coating can form almost all kinds of carbon coating. Guan et al. [22] first synthesized LFP/activated carbon composite with a size of 100–300 nm using the solvothermal method, mixed the composite with graphene oxide, and finally carried out a sol heat treatment to prepare an LFP/activated carbon/graphene composite, as shown in Figure 3b. The capacity increased from 105 mAh·g^−1^ in LFP to 156.3 mAh·g^−1^ in LFP/activated carbon and 167.3 mAh·g^−1^ in LFP/activated carbon/graphene. Furthermore, the capacity retention rate of LFP/activated carbon/graphene reached 96.3% after 300 cycles at a high rate of 30 C. Guo et al. [27] synthesized LFP/C composite materials via modification with carbon nanotubes (CNTs). The surface of LFP has a carbon coating layer with a thickness of 2–3 nm, and LFP/C nanoparticles are in close contact with CNTs, forming a three-dimensional network (Figure 3c). This coating treatment reduced the polarization effect from 332.7 mV in LFP to 63.3 mV in LFP/C and 45.6 mV in LFP/C/CNTs, indicating a significant improvement in the diffusion coefficient and conductivity of lithium ions. It should be noted that, although the carbon coating of LFP has achieved improvements in conductivity and rate performance, it sacrifices the compacted density of the battery and reduces the volume-specific energy density. To reduce the impact of the coating on the energy density of nanoscale electrodes, the coating process should be improved to prepare thinner and denser nano-thickness carbon layers in the future.

## 4. NCM Batteries

### 4.1. NCM Structure and Properties

**Figure 4:**
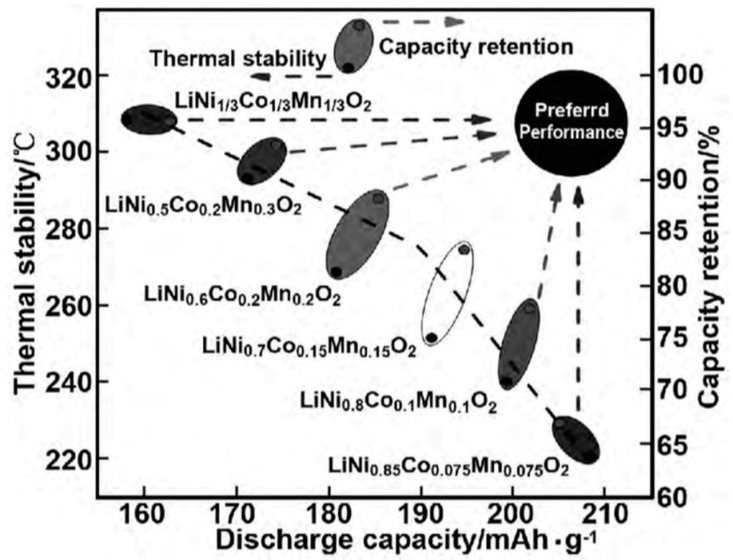
Discharge-specific capacity, thermal stability, and capacity retention of Ni, Co, and Mn transition metal-doped NCM [28].

NCM has a layered structure, and Li^+^ can diffuse in a layered two-dimensional plane, resulting in a high diffusion rate. Li^+^ occupies the *3a* position of the (1,1,1) crystal plane, transition metal (TM) ions occupy the *3b* position, and O^2+^ occupies the *6c* position. The TM layer can be randomly occupied by Ni, Co, and Mn, and each TM atom is surrounded by six oxygen atoms to form an octahedral structure. During the charging and discharging process, Li^+^ reversibly embeds and detaches between layers, and different TMs play a synergistic role: (1) Ni is an active substance, and during the charging and discharging process, Ni^+^/Ni^3+^ and Ni^3+^/Ni^4+^ pairs provide capacity. The higher the Ni content, the higher the capacity of the NCM battery. However, due to the similar radii of Li^+^ and Ni^2+^, the mixing of Li^+^ and Ni^2+^ ions intensifies with the increased Ni content. When the Ni content is higher than 80%, the cycle life of the NCM rapidly decays. As shown in Figure 4, with the increase in Ni content, both the thermal stability and capacity retention rate decrease while the capacity increases; (2) Co can effectively stabilize the layered structure of NCM, inhibit cation mixing, improve the conductivity of the material, and improve cycling performance; (3) Mn can reduce costs and improve the structural stability and safety of materials. Li_x_Ni_y_Mn_z_Co_(1−y−z)_O_2_ has a diverse composition, and the electrochemical performance can be further improved via doping, which has broad development prospects.

### 4.2. Life Attenuation Mechanism of NCM Battery

High-nickel NCM materials possess high specific capacity but still exhibit a series of problems, including (1) mechanical damage caused by phase transition during cycling and the loss of lattice oxygen and (2) the surface and internal crack surface of particles reacting with air and electrolyte to form a passivation film, reducing the conductivity and ion diffusion rate, and causing structural damage to the electrode material due to expansion [29].

#### 4.2.1. Mechanical Damage of NCM Particles

**Figure 5:**
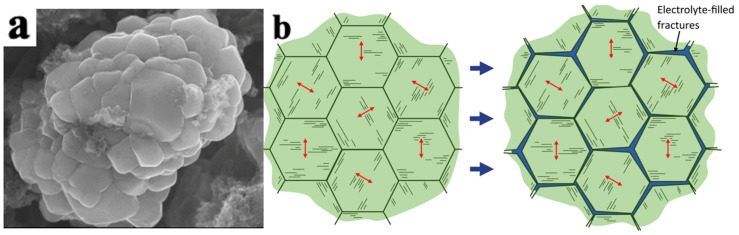
Particle morphology and cracks in LiNi_0.5_Mn_0.3_Co_0.2_O_2_ particles. (**a**) SEM morphology of LiNi_0.5_Mn_0.3_Co_0.2_O_2_ secondary and primary particles, (**b**) schematic diagram of the formation of intergranular cracks caused by anisotropic strain of layered primary particles within secondary particles. The arrows indicate the *c*-axis direction [30].

As shown in Figure 5, LiNi_0.5_Mn_0.3_Co_0.2_O_2_ secondary particles are composed of many primary particles. Accordingly, the mechanical damage of the electrode particles can be divided into the intragranular damage of the primary particles and the intergranular damage of the secondary particles.

**Figure 6:**
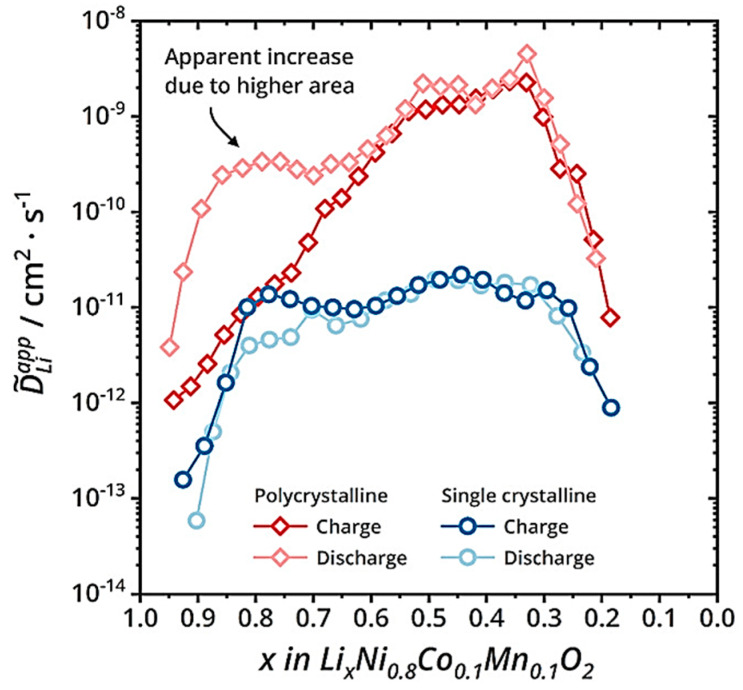
Comparison of lithium-ion diffusion coefficients between secondary and primary particles during charging and discharging processes [31].

The secondary particles shorten the propagation path of Li^+^ and electrons, thereby improving the rate performance of the electrode. As shown in Figure 6, the trend of Li^+^ changes in secondary and primary particles is roughly the same, which can be divided into three stages, including the growth stage, stable stage, and decrease stage. The reason for the increase in the diffusion coefficient in the first stage can be attributed to the increase in the concentration of lithium vacancies. The decrease in the diffusion coefficient in the third stage is due to the decrease in crystal interlayer spacing (Li^+^ diffusion channel) when the lithium removal reaches about 70%, and the crystal structure transitions to H3. Whether during charging or discharging, the diffusion coefficient of Li^+^ is much higher than that in primary particles. At the same time, nanoscale primary particles have serious side reactions and high production costs. Therefore, at present, electrode materials for electric vehicle power batteries are mainly secondary particles.

##### Intergranular Damage

**Figure 7:**
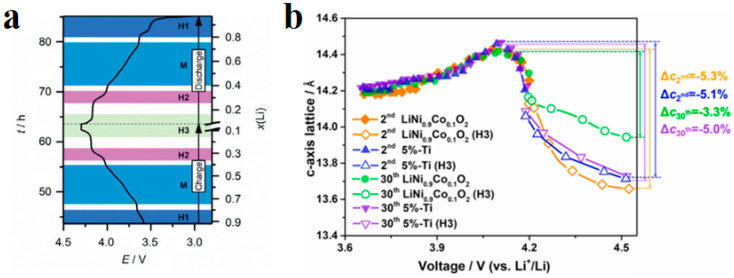
Primary particle intergranular cracks and lattice change during cycling process in NCM. (**a**) Crack propagation morphology between NCM primary particles [33]; (**b**) effect of Ti doping on the rate of lattice change in the *c*-axis during cyclic process [34].

Intergranular damage occurs in secondary particles and is caused by differences in the volume change between different phases. During the process of lithium removal, the crystal structure of NCM undergoes a series of H1-M-H2-H3 phase transitions, and the phase composition is closely related to the degree of lithium removal [32]. In the NCM delithium process, the first hexagonal phase observed is labeled H1, while the second hexagonal phase is labeled H2, and so on. The observed monoclinic phase is indicated by M. Xu et al. [33] measured the phase composition of Li_x_NiO_2_ and found that the phase composition is closely related to the voltage plateau and the amount of lithium removed. In detail, upon charge, pristine Li_0.99_Ni_1.01_O_2_ first undergoes a solid–solution reaction until ≈Li_0.8_Ni_1.01_O_2_, after which it exhibits a phase transformation from a hexagonal (H1 phase) to a monoclinic structure (M phase). The material maintains its monoclinic symmetry on further delithiation until ≈Li_0.4_Ni_1.01_O_2_. Then, it exhibits another phase transformation, namely M to H2 between x = 0.40 and 0.36 (x represents the lithium content, i.e., Li_x_TMO_2_). The H2 phase remains until x = 0.26. Finally, the range of x = 0.26–0.16 is associated with the first-order H2–H3 transition at ≈4.2 V. Due to the fact that the parameters of the H2 phase on the *c*-axis are much smaller than those of the H3 phase, it is widely believed that intergranular cracks are caused by the H2-H3 phase transition [34]. The arrangement of primary particles is random, and the internal stress generated by the contraction and expansion of adjacent primary particles acts on the grain boundaries. During the charging and discharging cycles, intergranular cracks expand, as shown in Figure 7a. The Coulombic efficiency is usually between 70 and 95% during the first cycle of the battery. On the one hand, this is due to the formation of a passivation layer on the electrode–electrolyte surface, which consumes some active lithium. On the other hand, this is due to intergranular damage caused during the first cycle [35]. Feng et al. [36] measured the lattice change rate on the *c*-axis after 2 and 30 cycles of undoped LiNi_0.9_Co_0.1_O_2_ (UM) and LiNi_0.9_Co_0.1_O_2_ (PM) doped with 5% Ti, as shown in Figure 7b. After two cycles, the change rate of the PM lattice on the *c*-axis was −5.1%, and the change rate of UM was −5.3%. After 30 cycles, the lattice change rate of the PM on the c-axis changed from −5.1% to −5.0%, and the lattice change rate of the UM on the *c*-axis changed from −5.3% to −3.3%. The lattice change rate on the *c*-axis significantly decreased during 2–30 cycles, indicating that the strain caused during the first cycle decays during the following cycles and the H2-H3 phase transition has a certain degree of reversibility.

**Figure 8:**
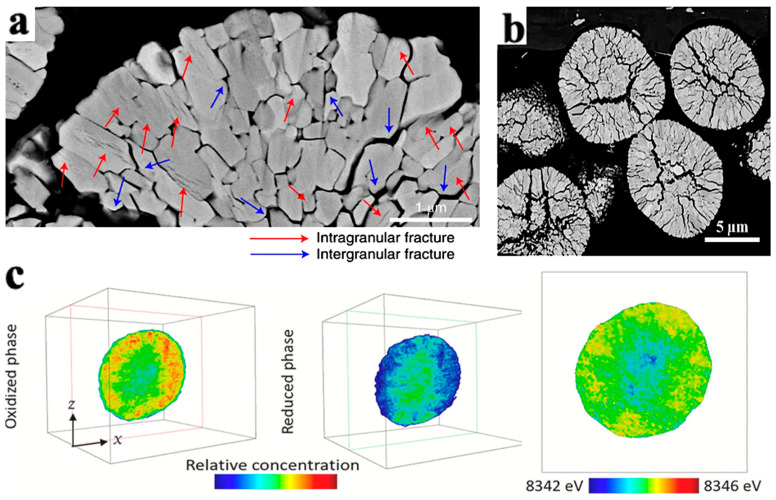
Crack morphology and particle chemical phase diagram caused by NCM phase transition. (**a**) SEM cross-sectional crack morphology of LiNi_0.89_Mn_0.1_Mo_0.01_O_2_ particles after 1000 cycles in the voltage range of 3.0–4.2V [37]; (**b**) SEM cross-sectional crack morphology of LiNi_0.88_Mn_0.1_Mo_0.02_O_2_ particles after the first cycle [38]; (**c**) chemical phase diagrams of oxidation and reduction phases in LiNi_0.8_Co_0.15_A1_0.05_O_2_ [36].

In addition, the cracks in secondary particles experience gradual propagation from the center to the surface during cycling, as shown in Figure 8a,b. This phenomenon is related to the SOC and phase transition during the extraction of Li^+^ process [35]. Deng et al. [36] took the chemical phase diagram of LiNi_0.8_Co_0.15_A1_0.05_O_2_ (NCA) particles, as shown in Figure 8c. In the diagram, the red part has strong activity, the blue part has weak activity, and the outermost and innermost redox activities of the secondary particles are the weakest. The weak activity of the outer layer is caused by a series of side reactions between the electrolyte and the surface of secondary particles, which reduces the oxidation–reduction activity of nickel on the surface of particles. 

The weak activity of the inner layer is due to cracks inside the secondary particles (the material at the center of the particles has a low degree of lithiation, and the SOC of the inner layer is lower than that of the outer layer, resulting in internal stress and the formation of cracks). In addition, the H3-H2 phase transition during the lithium intercalation process can also explain the phenomenon of cracks originating from inside the particles: the electrode particles after complete lithium removal are H2 phase. As the lithium intercalation process progresses, the H3 phase gradually forms from the surface of the particles inward, forming a core–shell structure. Since the parameters of the H2 phase inside the core–shell structure on the c-axis are much smaller than those of the external H3 phase, the internal stress in the H2 phase leads to the formation of cracks. When the crack extends to the surface of the particle and forms a penetrating crack, the electrolyte may enter the particle and undergoes interfacial reactions, resulting in an increase in the internal resistance of the electrode material. The volume expansion of the electrode–electrolyte interface can also lead to particle breakage.

##### Intragranular Damage

The lattice strain occurs in the cathode particles during the lithium intercalation cycle and the shrinkage and expansion cycle lead to the intragranular damage of the primary particles. Compared with secondary particles, primary particles are less prone to damage due to their micron size. The stress gradient inside and outside the particles is small, making it difficult to generate cracks internally. Moreover, propagation only occurs when the crack size is greater than the critical size. Therefore, it is difficult for small-sized primary particles to reach the critical size required for crack propagation. A comparison of the strain energy inside the particle and the fracture surface energy (2*γ*) can be used as a criterion for evaluating the critical size of particles. Tao et al. [39] proposed a method for predicting whether cracks can be stable within a single crystal using an induced stress model, as shown in Equation (1):

(1) $$\prod_{T_{p}}=\int \frac{\sigma^{2}}{2E}dV=\pi \times h\times {\left[\frac{\alpha \times E_{0}\times \left(C_{R}-C_{0}\right)}{1-\nu }\right]}^{2}\times \int_{0}^{r}\xi^{2}\frac{1}{E}\mathrm{rdr}<2\gamma$$

where *h* is the height of the cylindrical particle, *α* is the concentration expansion coefficient, *E*_0_ is the Young’s modulus of non-lithiated particles, *E* is the Young’s modulus at a given lithium-ion concentration, *C_R_* is the surface lithium-ion concentration, *C*_0_ is the center lithium-ion concentration, *ν* is Poisson’s ratio, *ξ* represents dimensionless stress, and *γ* is the surface energy.

Enrico et al. [31] conducted charging experiments on the secondary and primary particles of LiNi_0.8_Mn_0.1_Co_0.1_O_2_ at different voltages. It was found that the secondary particles began to crack at 3.8 V, while the primary particles did not show significant changes until 4.2 V, indicating that the primary particles show much higher structural stability than the secondary particles. Therefore, the cathode particle mechanical damage mainly occurs within the secondary particles.

#### 4.2.2. Loss of Lattice Oxygen

**Figure 9:**
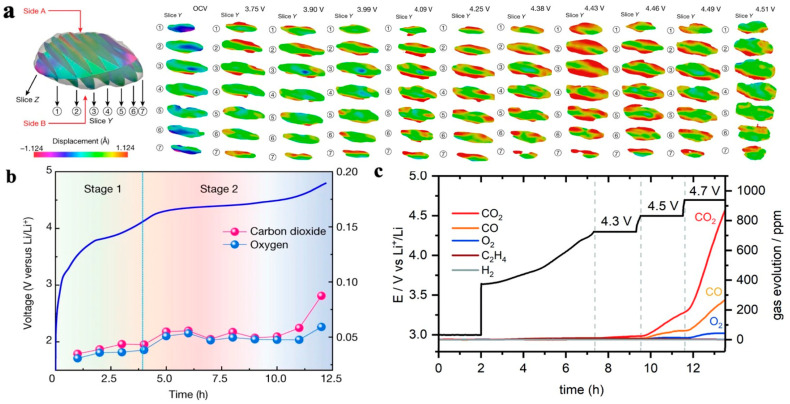
Release and gas production of lattice oxygen in NCM material. (**a**) Bragg coherent diffractive imaging (BCDI) of the particles in the strain field, with compressive strain in blue and tensile strain in red [40]; (**b**) electrochemical mass spectrometry of primary particles [40]; (**c**) time-dependent variation curve of gas production [42].

In electrode materials that are rich in transition metals, the lattice oxygen is unstable. When the degree of lithium removal in high nickel NCM is significant, the content of easily reducible high valence Ni is high. In order to balance the charge inside the transition metal layer, the TM-O bond may break and generate oxygen vacancies, which will detach in the form of gas and a loss of lattice oxygen. Liu et al. [40] recorded the stress–strain behavior of cathode particles at different voltages during the discharge process of LiNi_0.13_Mn_0.54_Mo_0.13_O_2_ electrode materials rich in Li and Mn using Bragg coherent diffractive imaging (BCDI) and electrochemical mass spectrometry. As shown in Figure 9a,b, in the first stage (3.75–4.43 V), there is no significant gas overflow related to oxygen. When the voltage increases to 4.43 V, the tensile strain of the particles reaches its maximum value, and the electrostatic repulsion force between the oxygen layers reaches its maximum, indicating that the cathode particles are almost completely de-lithium; in the second stage (4.43–4.51 V), the release of lattice oxygen significantly intensifies, and the particle tensile strain gradually decreases until the strain disappears at 4.51 V, indicating a close correlation between the cathode particle strain and gas production.

The release of lattice oxygen leads to structural degradation, capacity loss, and exacerbates side reactions, resulting in a series of safety issues. Clare et al. [41] studied the chemical decomposition of electrolytes on the LiNi_0.8_Mn_0.1_Co_0.1_O_2_ electrode via gas testing and nuclear magnetic resonance spectroscopy (NMR). The diversity of decomposition products generated on the positive side was clarified, and it was observed that the release rate of oxygen-containing-compounds changed with the voltage, as shown in Figure 9c. This fact indicates the diversity of gas production from the reaction between the cathode material of an NCM battery and the electrolyte, and that the gas production is closely related to the voltage platform.

### 4.3. Modification Methods for NCM

#### 4.3.1. Surface Coating Modification

As mentioned earlier, NCM material particles inevitably undergo structural damage during cycling. Typically, cracks propagate from the center to the surface of the particles. As a result, the electrolyte enters the interior of the cathode particles to form a passivation film, resulting in adverse effects such as particle volume expansion and reduced conductivity and Li^+^ diffusion rates. At present, coating is the most effective method to improve the cathode materials without changing the manufacturing technologies [43]. As shown in Table 2, the coating morphology on the particle surface can be divided into two types: homogeneous coating and island coarse coating. The coating morphology is directly related to the coating process. Through the use of vapor chemical technology (atomic layer deposition and chemical vapor deposition), a coating with a thickness of 0.1 nm can be prepared, and the coating thickness can be increased via successive deposition [44].

According to the data in Table 2, coatings can significantly improve cycling performance due to the following three reasons: (1) coatings can serve as supporting structures to alleviate stress caused by phase transformation and improve the structural stability of particles; (2) homogeneous coating can better form a physical barrier to reduce the occurrence of side reactions; and (3) the coating materials are mostly metal oxides, which can neutralize HF, reduce acidity, and reduce the migration of transition metals. The common metal oxide layers include ZnSnO_3_ [45], ZnAl_2_O_4_ [46], etc., and coated NCM shows a higher capacity retention rate than uncoated NCM. Although homogeneous coating is technically feasible, the application of homogeneous coatings in large-scale production is not feasible for power batteries due to the high cost. On the other hand, island-like coating shows great development prospects due to its economic efficiency. Island coatings can be prepared using both dry and wet processes, which are both operationally feasible and cost-effective. Compared with wet methods, the dry coating process is more cost-effective and environmentally friendly, which helps in the industrial production of power batteries [44].

**Table 2:** Homogeneous and island coarse coatings on cathode materials and the effects of coating modification on battery properties [47].

Morphology	Technique	Cathode Materials	Coating	Capacity (mAh·g^−1^)/Retention Rate (%) (Rate C/Cycle Times)		Ref.
				Before Coating	After Coating	
Homogeneous coating 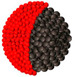	Gas phase chemical coating	Li_1.2_Mn_0.54_Ni_0.13_Co_0.13_O_2_	Al_2_O_3_	251–232/92.43% 0.05 C/30	271–257/94.83% 0.05 C/30	[48]
		NCM523	ZrO_2_	216–84/40% 0.03 C/100	228–182/83.25% 0.03 C/100	[49]
		Li_1.17_Mn_0.48_Ni_0.23_Co_0.12_O_2_	MgO	240–234/97.9% 0.1 C/10	260–258/99.55% 0.1 C/10	[50]
Island coarse coating 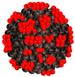	Dry coating	LiNi_0.815_Co_0.15_Al_0.035_O_2_	Li_3_PO_4_	195–139/70.55% 1 C/100	192–171/89.06% 1 C/100	[51]
		NCM525	Li_2_MoO_4_	186–97 48% 0.2 C/50	178–138 78% 0.2 C/50	[52]
	Wet coating	Li[Li_0.05_Ni_0.4_Co_0.15_Mn_0.4_]O_2_	Al_2_O_3_	155–133/86% 1 C/50	157–150.7/96% 1 C/50	[44]
		NMC532	Li_3_PO_4_	135–44/32.59% 1 C/100	214–189/88.32% 1 C/100	[53]

#### 4.3.2. Elemental Doping Modification of NCM

The volume change and gas generation of NCM are closely related to their cyclic phase transition. Another effective method to suppress phase transition is bulk doping. According to the classification of doping sites, it can be divided into Li-site doping, TM-site doping, and O-site doping. Different doping sites also have different improvements in material properties [54]. Compared with the LFP olivine structure, the stability of the NCM-layered structure is relatively poor, and the composition is more diverse. Therefore, doping has a more significant impact on the performance of NCM. Among them, Li-site doping can not only increase vacancies but also reduce the mixing of Li/Ni, preventing Ni migration. Due to the introduction of elements with higher valence states than Li^+^, the valence state of Ni will decrease according to the conservation of valence states. Furthermore, constant valence metal elements, such as Mg, will generate pillar effects in the lattice, thereby improving the stability of the structure. Introducing stronger TM-O bonds (such as Al, Ti, Zr, etc. [44]) at the TM site can effectively stabilize the crystal structure, prevent lattice oxygen loss, and suppress harmful H2-H3 phase transitions, thereby improving thermal stability and cycle life. 

For high nickel NCM, the main purpose of atom doping is to prevent the mixing of nickel atoms. Wang et al. [55] used Mg^2+^ and Ti^4+^ to replace the Ni^2+^ position in LiNi_0.83_Co_0.11_Mn_0.06_O_2_, which can effectively prevent Ni atoms from entering the Li position. For low nickel NCM, the rate performance is improved mainly by anion doping. W-doped LiNi_1/3_Co_1/3_Mn_1/3_O_2_ shows excellent high-rate performance synthesized via hydrothermal lithiation [56]. Of course, proper atom doping can improve the capacity retention rate by changing the bond strength, but excessive atom doping can lead to structural distortion and inhibit the cyclic stability of the cathode [57].

## 5. Safety of Power Batteries

In the field of power batteries, safety is a measure of the risk of personal injury. The battery safety test items specified in GB 38031 [58] include external short circuits at normal temperature, overcharge, overdischarge, low pressure tests, temperature cycle, extrusion, acupuncture, etc. The most important thing for battery safety is to prevent thermal runaway. If the temperature of the battery rises beyond the critical thermal runaway temperature, it may cause the combustion or even explosion of the adjacent battery pack [59]. The transition temperatures (from lamellar phase to spinel phase) of NCM433, NCM532, NCM622, and NCM811 are 245 °C, 235 °C, 185 °C, and 135 °C, respectively [60]. The temperature range of the spinel phase decreases gradually with the increase in Ni content, corresponding to the reduced thermal stability of NCM. In addition, NCM phase transition often occurs in the surface layer of the particles, accompanied by the release of oxygen atoms. The released oxygen may react with the electrolyte to produce a large amount of heat and gas, which further deteriorates battery safety. On the contrary, LFP does not show significant weight loss at temperatures below 230 °C and has better thermal stability, which is attributed to the fact that Fe-P-O bonds in olivine-structure LFP are much stronger than Ni-O, Co-O and Mn-O bonds in layer-structured NCM [61]. Liu et al. [62] confirmed that LFP batteries exhibit obvious thermal stability advantages over NCM batteries by comparing the initial heat release temperature, maximum heat release rate, maximum temperature, and heat release time. On the other hand, the thermal stability of the battery for the same system is greatly related to the SOC. The higher the SOC, the worse the thermal stability and safety of the battery.

It should be noted that volatile and flammable liquid electrolytes are a restricting factor in terms of the safety of power batteries. Replacing traditional liquid electrolytes with solid electrolytes sidesteps many of the safety issues associated with traditional LIBs. Yu et al. [63] found that in the event of a fire solid-state batteries with only a small amount of liquid released about one-fifth of the heat of traditional LIBs, while solid-state batteries with pure solids produced little heat. Of course, all-solid-state batteries are technically challenging, especially to form a stable and good solid–solid interface between the cathode and electrolyte. First, all-solid-state batteries require a smaller volume expansion of the cathode during charging and discharging. Jung et al. [64] fabricated LiNi_0.75_Co_0.10_Mn_0.15_O_2_ with rod-like grains, which enabled the cathode to adapt to volume changes, maintain its mechanical integrity, and enhance its reversible ability. In addition, the interface should have good chemical and electrochemical stability. Deng et al. [65] deposited semiconductor poly(3,4-ethylenedioxythiophene) on the surface of NCM811 to inhibit the side reaction between the electrolyte and cathode. For sulfide solid electrolytes, the formation of a space charge layer with a low lithium-ion concentration at the interface should be avoided. Sun et al. [66] constructed in situ Li_3_P_1 + x_O_4_S coating on the surface and grain boundary of NMC811 material to increase the lithium-ion concentration at the interface of the cathode and electrolyte. This can help to solve the problem of the poor cycling stability of NMC811 cathode material in sulfide-based lithium batteries. On the other hand, compared with NCM, LFP has a lower operating voltage and better structural stability and thus shows a higher capacity retention rate in all-solid-state batteries [67].

## 6. Conclusions and Outlooks

This article reviews the material structures, performance-degradation mechanisms, and modification approaches of the two most commonly used power batteries, LFP and NCM. The impact of coating and doping modification on the performance has been summarized. The main conclusions are as follows:

- The olivine crystal structure of LFP resulted in its low conductivity and ion diffusion rate, leading to the partial deactivation of the cathode particles, a loss of active lithium, and a lower rate performance, limiting the charge and discharge rate in the battery.
- The LFP lithium removal exhibited significant heterogeneity. The FP phase is distributed in a fine filament shape and accompanied by regional condensation, leading to the polarization of the LFP/FP phases in the cathode particles. The uncoordinated polarization behavior between the two phases induced internal stress within the particles, leading to cracks and structural damage in the particles.
- The size of the first-order particles in the NCM materials affected the generation of cracks during their cycling process. When the first-order particle size was smaller than the crack initiation critical size, internal cracks in the first-order particles were hard to initiate and propagate.
- The H2-H3 phase transition can induce cracks in the secondary particles during the cycling of the secondary particles in the NCM materials. The electrolyte may enter the interior of the particles through microcracks and form a passivation film on the surface of the cracks, increasing the volume of the particles and causing breakage of the particles.
- The phase transition of the NCM materials induced lattice oxygen release and structural degradation. In addition, various gases, such as CO_2_, CO, O_2_, H_2_, and C_2_H_4_, can be generated, causing safety issues and structural damage.

Based on the performance degradation principle of LFP and NCM materials, in-depth research works are needed in the field of composition design, material nanosizing, coating, and doping to further improve battery performance and solve the bottleneck problems of life degradation. The main approaches include the following:

- The nanosizing and coating of cathode materials need to be applied simultaneously to improve the conductivity and ion diffusion rate and reduce side reactions at the electrode electrolyte interface. For LFP, its interfacial conductivity can be improved through the use of coatings, such as carbon coatings, which show a good coating effect and economic benefits. However, for NCM materials, the coating material needs to serve as a support and physical barrier, requiring careful control of the type and morphology of the coating material. At present, it is still difficult to achieve a thin and uniform coating on the surface of NCM on the basis of low cost, which affects the vibration density and energy density of electrode materials. Further development of coating processes is needed for LFP and NCM to reduce coating costs and increase energy density.
- The main purpose of LFP doping is to improve the conductivity and ion diffusion rate of the material, while the main purpose of NCM material doping is to suppress phase transition. Current research is mostly focused on single-atom doping. Due to the limited performance improvement of single-atom doping, further research is needed on multiple-atom co-doping, elucidating the doping ratios and synergistic effects of multiple doped atoms, and seeking low-cost doping processes.

## Data Availability

The data are available from the corresponding authors upon reasonable request.
